# Supplementary material for: Integrated digital pathology and transcriptome analysis identifies molecular mediators of T-cell exclusion in ovarian cancer
Source: Nat Commun. 2020 Nov 4;11:5583. doi: 10.1038/s41467-020-19408-2 (PMC7642433; doi:10.1038/s41467-020-19408-2)
Supplement: Supplementary file 1 — Supplementary Information [file 41467_2020_19408_MOESM1_ESM.pdf]

Integrated Digital Pathology and Transcriptome Analysis Identifies Molecular Mediators of T-cell Exclusion in Ovarian Cancer

Mélanie Desbois†, Akshata R. Udyavar†, Lisa Ryner†, Cleopatra Kozlowski#, Yinghui Guan#, Milena Dürrbaum, Shan Lu, Jean-Philippe Fortin, Hartmut Koeppen, James Ziai, Ching-Wei Chang, Shilpa Keerthivasan, Marie Plante, Richard Bourgon, Carlos Bais, Priti Hegde, Anneleen Daemen, Shannon Turley, and Yulei Wang.

Supplementary Methods

Pseudo-code used with Definiens software for Digital Pathology analysis ....*Page 2*

Supplementary Figures

Supplementary Fig. 1. Identification of genes associated with CD8 quantity and/or CD8 spatial distribution using Random Forest and consensus clustering analysis ....*Page 3*

Supplementary Fig. 2. PAMR classifier analysis to derive a classifier for the prediction of the three immune phenotypes ....*Page 5*

Supplementary Fig. 3. Multivariate analysis of the tumour-immune phenotypes in association with the tumour stage, age and debulking status in the ICON7 cohort.........*Page 6*

Supplementary Fig. 4. Pathway enrichment analysis characterizing the 3 immune phenotypes ....*Page 7*

Supplementary Fig. 5. Gene-expression based molecular classifier for predicting the immune phenotypes in the vendor procured cohort ....*Page 8*

Supplementary Fig. 6. Additional data for on ovarian cancer cells and fibroblasts supporting the Fig. 5 ....*Page 10*

Supplementary Fig. 7. Survival analysis comparing infiltrated (intra-epithelial TILs) and combined excluded/desert tumours (no/low intra-epithelial TILs) ....*Page 11*

Supplementary Fig. 8. Demographic table for the ICON7 cohort ....*Page 12*

Supplementary Methods

Pseudo-code used with Definiens software:

Original images were scanned in NDPI format at 0.46um/pixel. RGB layers were stored as Layer 1 = Red, Layer 2 = Green, Layer 3 = Blue.

Images are first analysed at low magnification (0.2x).
Layer “filtered” = Gaussian blur (kernel 11x11) on Layer 1
"Tissue" region separated from "Background" at threshold 230 on Layer “filtered”.
Objects smaller than 5000 pixel2 were classified as the same as surrounding class.
"Tissue" region is tiled using “chessboard segmentation” at scale factor 30.

All tiles were analysed at full magnification (20x).
A check is performed to make sure tissue area occupies at least 100000 pixel2 in the full resolution tile. Otherwise the tile is not analysed.
Normalized layers are computed as follows:
Layer “brown” = 3x10^4 * Layer 1 / Layer 3 /(Layer 1 + Layer 2 + Layer 3)
Layer “blue” = 265 * Layer 3 /(Layer 1 + Layer 2 + Layer 3)

On image object level “Tissue level”
Layer “filtered blue” = Gaussian blur (kernel 101 x 101) on “blue”
"Stroma" was separated from "Tumour" based on “filtered blue” with threshold 86.

On a separate image object level “Cell level”
"Nuclei" are separated from "Background" using “mean blue” with threshold 100.
Positive staining nuclei and negative nuclei were separated using “mean brown” with threshold 80.
Stained nuclei with "brightness" (averaged intensity of 3 RGB layers) below 90 were categorized as ‘anthracosis’ artefact, and not counted as positive cells.

The number and areas of positive nuclei were reported relative to total stroma or epithelial area.

Supplementary Figures

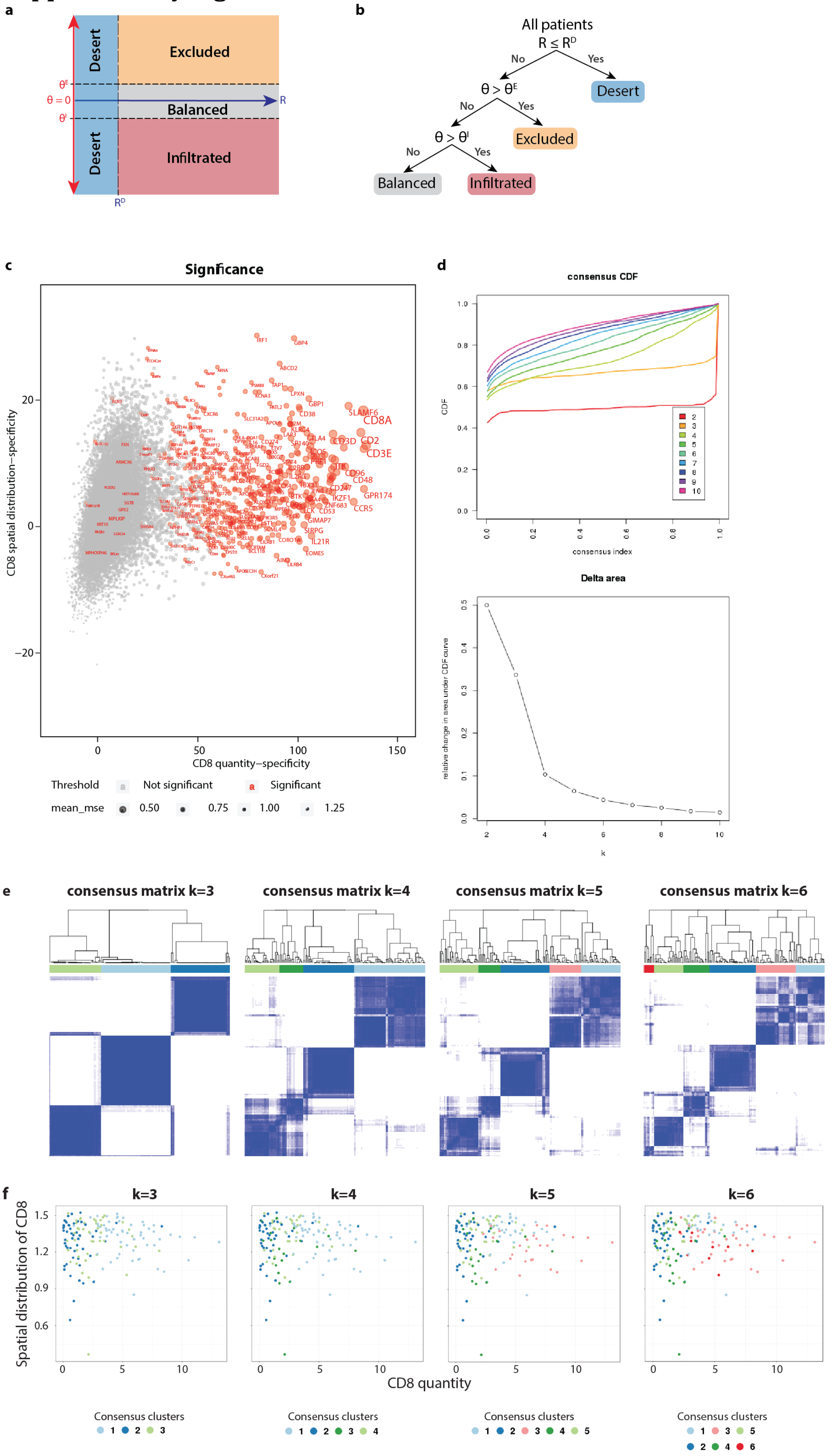

Supplementary Fig. 1. Identification of genes associated with CD8 quantity and/or CD8 spatial distribution using Random Forest and consensus clustering analysis. a-b Illustrations of the polar coordinates graph representing the expected distribution of immune phenotype based on R and θ metrics. **c** Random Forest was applied to tumour samples from the ICON7 clinical trial (n=155 training set). Genes with an average MSE above the 1st quantile were considered significant and are highlighted in red. **d-f** Consensus clustering analysis was performed on the ICON7 training set (n=155 samples). **d** (Top) Cumulative distribution function (CDF) of the consensus matrix for number of clusters k varying from 2 to 10 and (bottom) relative change in area under the CDF curve per increase in k by 1. **e** Heatmap illustrations display the consensus matrix for k from k=3 to k=6. **f** Two-dimensional representation of CD8 distribution with the tumour dots coloured by cluster for k from 3 to 6. Source data are provided as a Source Data file.

a

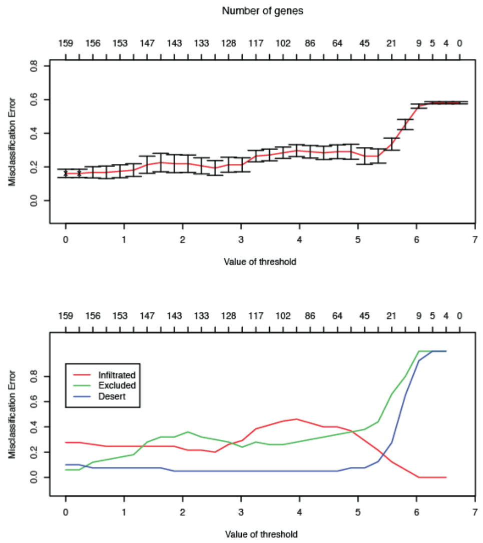

b

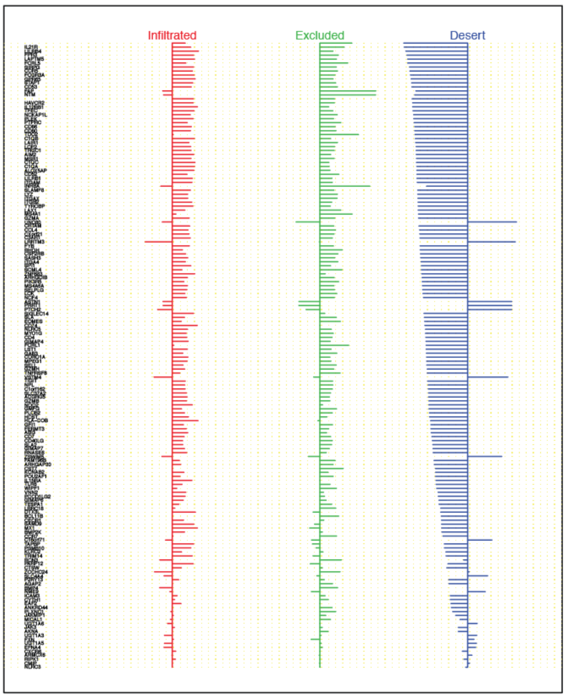

c

	Desert	Excluded	Infiltrated	Class error rate
Desert	36	3	1	0.1000000
Excluded	2	47	1	0.0600000
Infiltrated	12	6	47	0.2769231

Overall error rate = 0.161

d

	Pathologist/classifier (% concordance)
Infiltrated	20/42 (47%)
Excluded	28/31 (90%)
Desert	21/41 (51%)

Supplementary Fig. 2. PAMR classifier analysis to derive a classifier for the prediction of the three immune phenotypes. a Misclassification error rate overall (top) and per immune phenotype (bottom) for a PAM (Partitioning Around Medoids) classifier in function of number of classifier genes ranging from 159 to 1. The 95% confidence interval is depicted here (n=155 samples). **b** Centroids of the 159 entrez ids (157 genes) per immune phenotype. **c** Performance of the 157-gene immune phenotype classifier on the 155 training samples. Rows show true and columns predicted immune phenotypes. **d** Comparison of tumour-immune phenotype assignment by pathologist vs. gene classifier for 114 tumour cases of the ICON7 collection. Source data are provided as a Source Data file.

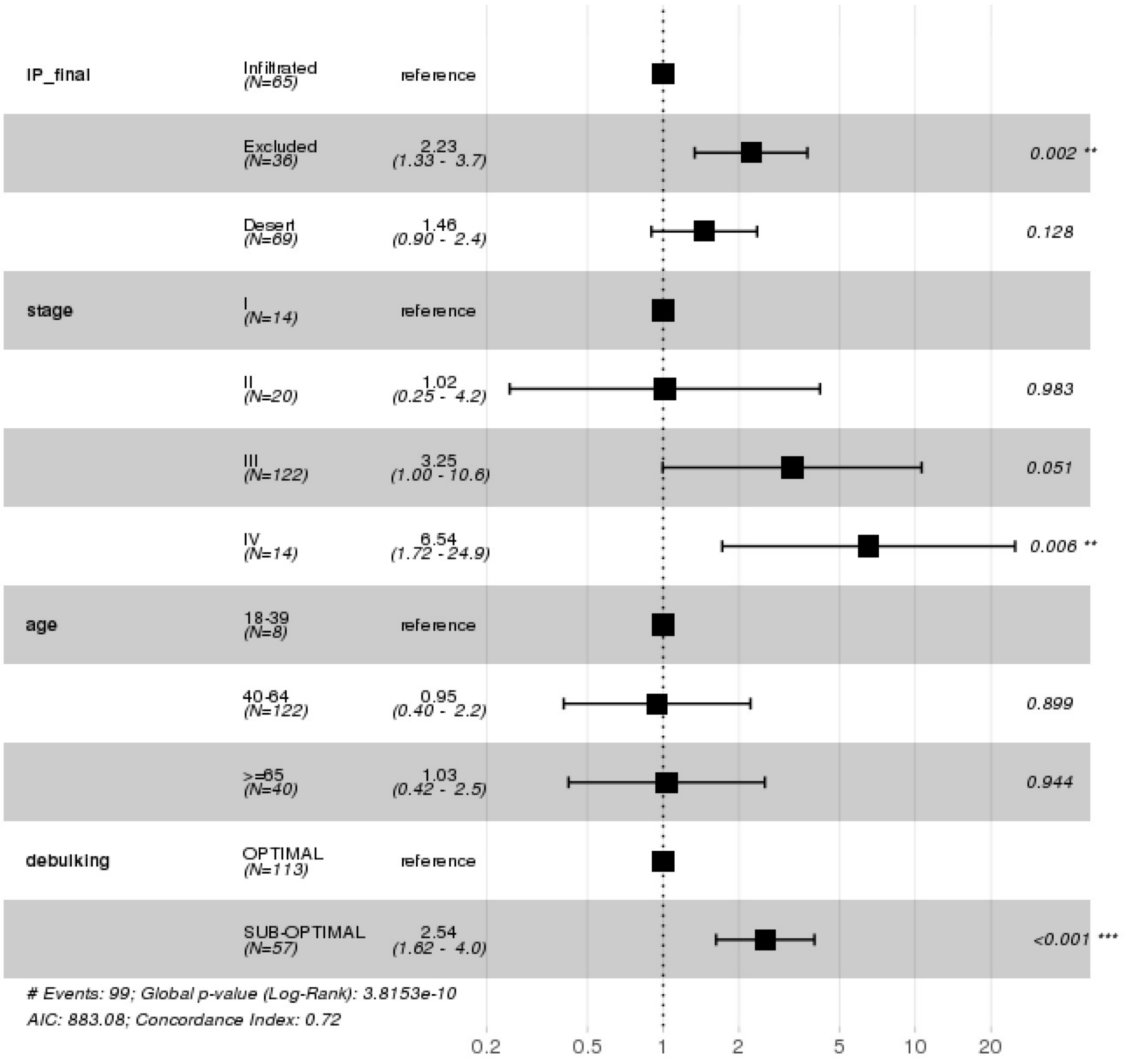

Supplementary Fig. 3. Multivariate analysis for the ICON7 cohort. Association of the tumour-immune phenotypes with PFS in a multivariate analysis correcting for the tumour stage, age and debulking status in the ICON7 cohort (control chemo arm only). The error bars represent the 95% confidence intervals of the hazard ratio (Cox Proportional-Hazards Model, no multiple testing). Source data are provided as a Source Data file.

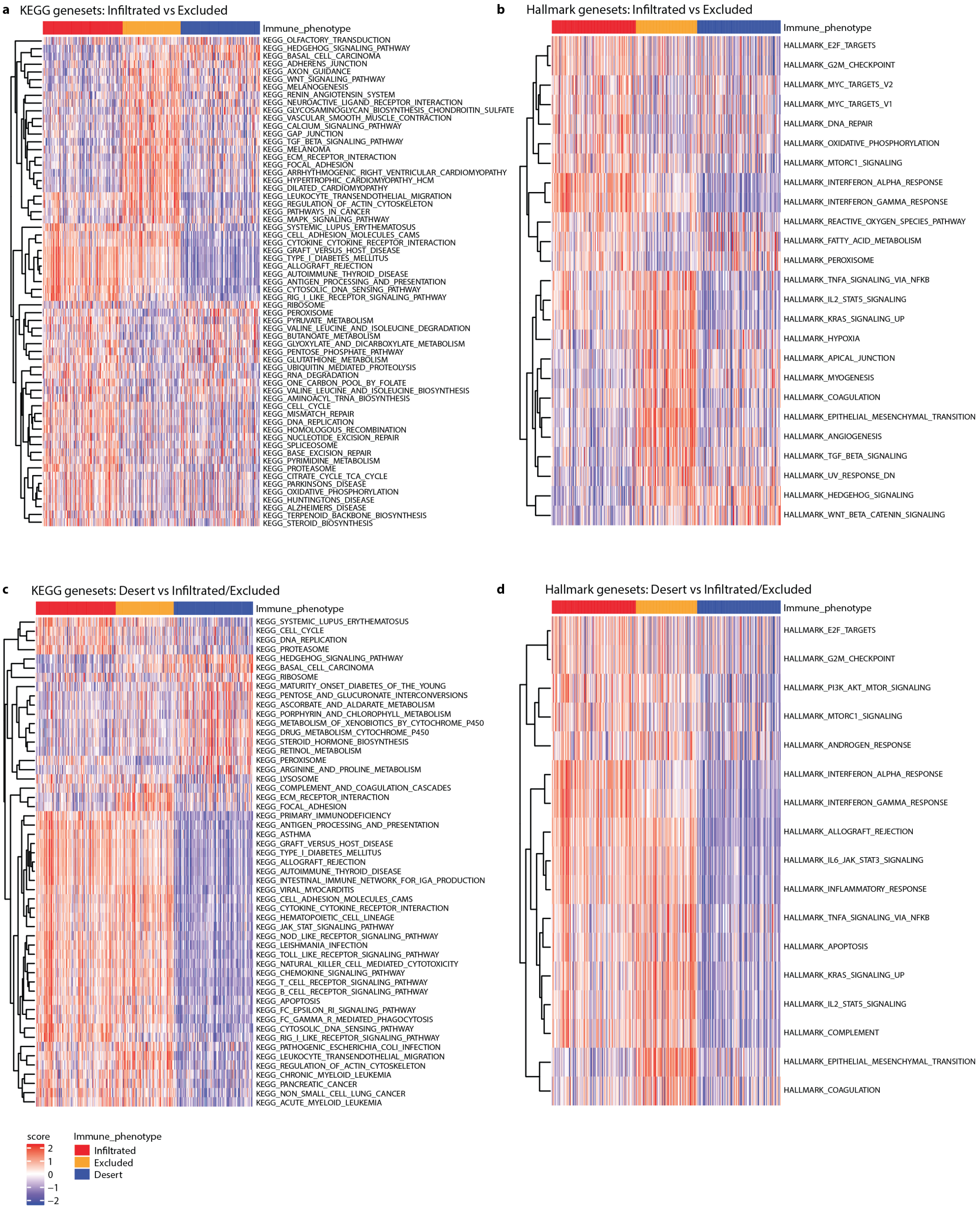

Supplementary Fig. 4. Pathway enrichment analysis characterizing the 3 immune phenotypes in the ICON7 cohort. Heatmap illustrations with average pathway-level z-scored expression for significant (FDR < 0.1) **a-b** KEGG and Hallmark pathways respectively from the infiltrated vs excluded comparison (n=351 samples), and **c-d** KEGG and Hallmark pathways from the desert vs infiltrated/excluded comparison (n=351 samples) supporting Figure 3b.

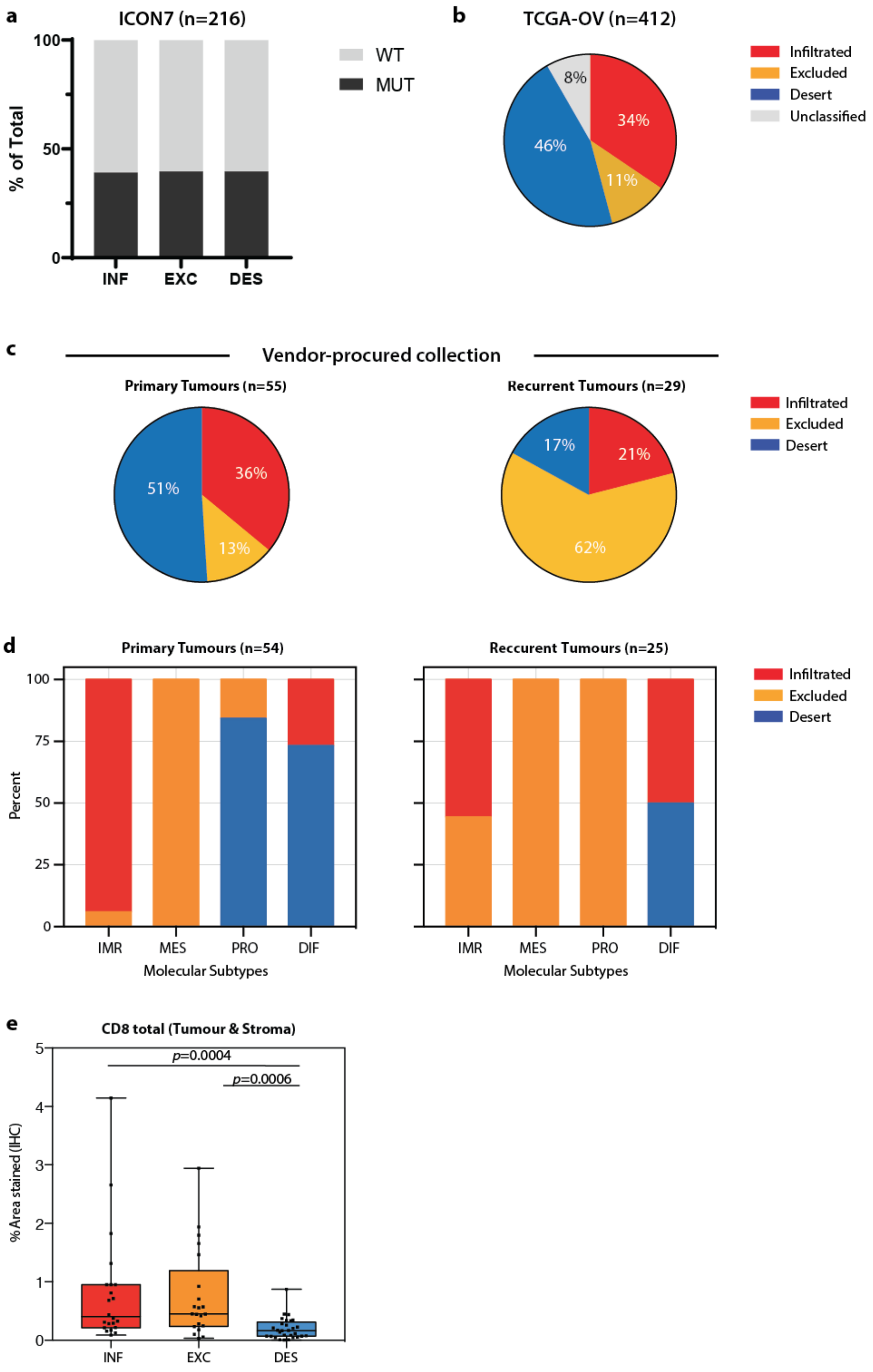

Supplementary Fig. 5. Gene-expression based molecular classifier for predicting the immune phenotypes in the vendor procured cohort. a 216 ovarian cancer samples had both mutation data and characterized immune phenotypes. The graph depicts % of mutation (MUT) or wild type (WT) in each tumour-immune phenotype. **b** Distribution of the predicted immune phenotypes based on gene expression in the TCGA-OV collection (n=412 samples). Red = Infiltrated, orange = Excluded, blue = Desert and grey = unclassified samples. **c** Distribution of

the predicted immune phenotypes based on gene expression the vendor procured collection for the primary tumours (n=55 samples) (left) and recurrent tumours (n=29 samples) (right). **d** Association of the immune phenotypes and molecular subtypes in the vendor procured collection. Left, primary tumours (n=54 samples); right, recurrent tumours (n=25 samples). Each bar displays the percentage of tumours of a particular molecular subtype classified as infiltrated, excluded or desert. Five samples that did not have molecular subtype labelling, including 1 primary and 4 recurrent tumours, were excluded from the analysis. IMR: Immunoreactive; MES: Mesenchymal; PRO: Proliferative; DIF: Differentiated. **e** The percent of both tumour and stroma areas stained for CD8 over the entire tumour (Tumour epithelium and stroma) is displayed (n= 72 samples). Whiskers ranging from minima to maxima, median and 25–75% IQR shown by box plots. The statistical significance is calculated using the Kruskal-Wallis test with a Dunn's multiple comparison test and the *p*-values are depicted on the graph. Source data are provided as a Source Data file.

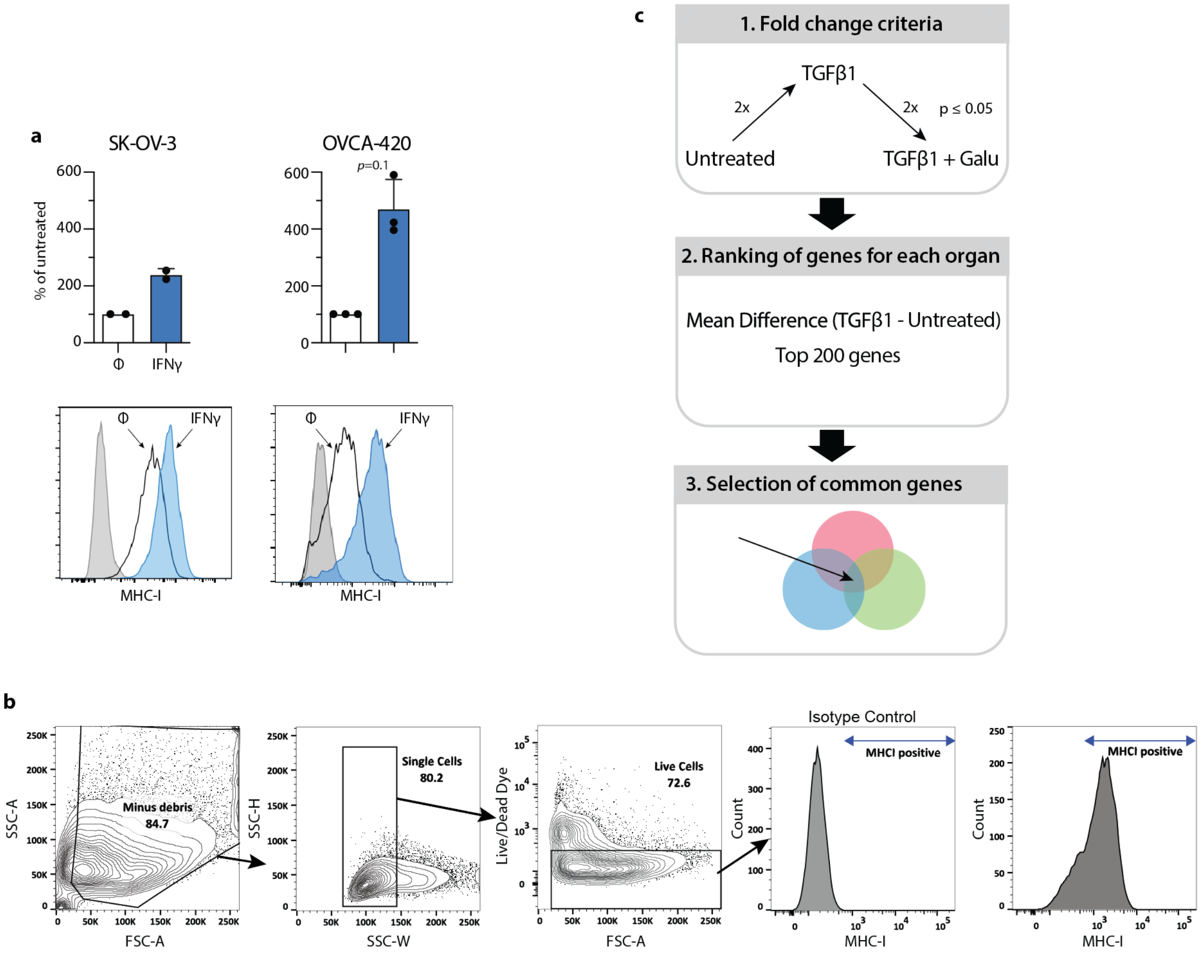

Supplementary Fig. 6. Multi-faceted role of TGFβ in ovarian cancer tumour microenvironment. a The expression of surface MHC-I (HLA-ABC antibodies) after IFNɣ treatment was analysed on SK-OV-3 and OVCA-420 ovarian cancer cell lines by flow cytometry. (Top) The box plots display the percentage of change compared to untreated cells for n=2 (SKO-V-3) or 3 (OVCA-420) independent experiments. Error bars, mean ±SD. Statistics are calculated with a Mann-Withney two-sided test and *p*-value is displayed on the graph. (Bottom) Flow cytometry images from one experiment are shown. Grey: isotype control, black line: untreated and blue: IFNɣ-treated cells. **b** Gating strategy to analyse MHC-I expression in ovarian cancer cell lines. **c** Schematic workflow illustrating criteria applied to identify the 77 common genes up-regulated upon TGFβ1 in the three normal primary fibroblasts. Source data are provided as a Source Data file.

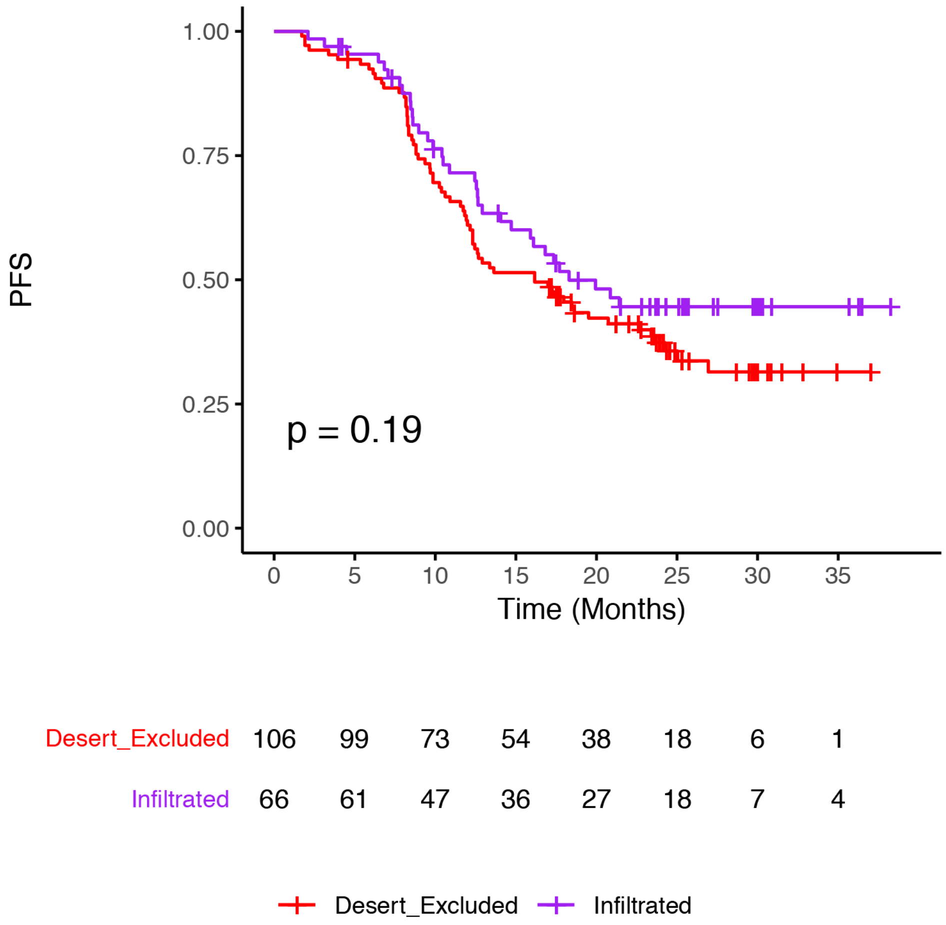

Supplementary Fig. 7. Association of the progression free survival with the tumour-immune phenotype, comparing infiltrated or excluded/desert tumours. The graph includes patients in the chemo control arm for the ICON7 cohort (n=172 patients). *p*-value is generated from a Cox Proportional-Hazards Model, no multiple testing. Source data are provided as a Source Data file.

	CARBOPLATIN PLUS PACLITAXEL CHEMOTHERAPY	CARBOPLATIN PLUS PACLITAXEL CHEMOTHERAPY PLUS BEVACIZUMAB
**n**	182	188
**Histology**	**% (n)**	**% (n)**
Serous	71 (130)	69 (130)
Clear cell	10 (18)	13 (25)
Endometrioid	9 (16)	5 (9)
Mucinous	2 (3)	1 (2)
Serous/Clear Cell/Endometrioid/ Mucinous/Other Mixed	5 (9)	9 (16)
Endometrioid/Clear cell/Mixed	1 (1)	2 (3)
Papillary Cystoadenocarcinoma/Unclassified/ Undifferentiated/Adenocarcinoma	3 (5)	2 (3)
**Original cancer**	**% (n)**	**% (n)**
Ovary (epithelial)	90 (164)	88 (165)
Fallopian tube	2 (4)	3 (5)
Primary peritoneum	5 (10)	7 (14)
Mixed	2 (4)	2 (4)
**Age Group**	**% (n)**	**% (n)**
18-39yr	4 (8)	3 (5)
40-64yr	72 (131)	76 (144)
>=65	24 (43)	21 (39)
**FIGO Stage**	**% (n)**	**% (n)**
Stage I	8 (14)	8 (15)
Stage II	11 (20)	14 (26)
Stage III	72 (131)	68 (127)
Stage IV	9 (17)	11 (20)
**Grade**	**% (n)**	**% (n)**
1	5 (10)	4 (8)
2	16 (30)	16 (31)
3	77 (141)	79 (148)
unknown	1 (1)	1 (1)
**Platinum Sensitivity**	**% (n)**	**% (n)**
Sensitive	49 (89)	62 (117)
Intermediate	19 (35)	20 (37)
Resistant	27 (50)	16 (30)
Refractory	1 (2)	0 (0)
NA	3 (6)	2 (4)
**Baseline CA-125 Category**	**% (n)**	**% (n)**
< 2x ULN	49 (89)	39 (74)
>= 2x ULN	50 (92)	60 (112)
NA	1 (1)	1 (2)

Supplementary Fig. 8. Demographic table detailing the histology, origin, age, tumour stage and grade, platinum sensitivity and CA-125 biomarker expression in the ICON7 cohort (n=370). Specimens are split based on the treatment received by the patient. Source data are provided as a Source Data file.
