# Supplementary material for: Integrated digital pathology and transcriptome analysis identifies molecular mediators of T-cell exclusion in ovarian cancer
Source: Nat Commun. 2020 Nov 4;11:5583. doi: 10.1038/s41467-020-19408-2 (PMC7642433; doi:10.1038/s41467-020-19408-2)
Supplement: Supplementary file 3 — Descriptions of Additional Supplementary Files [file 41467_2020_19408_MOESM3_ESM.pdf]

Descriptions of Additional Supplementary Files

Supplementary Dataset 1

Description: Table depicting genes associated with CD8 spatial distribution and/or CD8 quantity.

Supplementary Dataset 2

Description: Tumour-immune phenotypes prediction of the TCGA-OV dataset.

Supplementary Dataset 3

Description: R file as a source data for PAMR classifier.
