# Supplementary material for: Integrated digital pathology and transcriptome analysis identifies molecular mediators of T-cell exclusion in ovarian cancer
Source: Nat Commun. 2020 Nov 4;11:5583. doi: 10.1038/s41467-020-19408-2 (PMC7642433; doi:10.1038/s41467-020-19408-2)
Supplement: Supplementary file 7 — Reporting Summary [file 41467_2020_19408_MOESM7_ESM.pdf]

nature research

Corresponding author(s): Yulei Wang
Last updated by author(s): Sep 21, 2020

Reporting Summary

Nature Research wishes to improve the reproducibility of the work that we publish. This form provides structure for consistency and transparency in reporting. For further information on Nature Research policies, see Authors & Referees and the Editorial Policy Checklist .

Statistics

For all statistical analyses, confirm that the following items are present in the figure legend, table legend, main text, or Methods section.

n/a	Confirmed	
☐	☒	The exact sample size (*n*) for each experimental group/condition, given as a discrete number and unit of measurement
☐	☒	A statement on whether measurements were taken from distinct samples or whether the same sample was measured repeatedly
☐	☒	The statistical test(s) used AND whether they are one- or two-sided<br>*Only common tests should be described solely by name; describe more complex techniques in the Methods section.*
☐	☒	A description of all covariates tested
☐	☒	A description of any assumptions or corrections, such as tests of normality and adjustment for multiple comparisons
☐	☒	A full description of the statistical parameters including central tendency (e.g. means) or other basic estimates (e.g. regression coefficient) AND variation (e.g. standard deviation) or associated estimates of uncertainty (e.g. confidence intervals)
☐	☒	For null hypothesis testing, the test statistic (e.g. *F*, *t*, *r*) with confidence intervals, effect sizes, degrees of freedom and *P* value noted<br>*Give P values as exact values whenever suitable.*
☒	☐	For Bayesian analysis, information on the choice of priors and Markov chain Monte Carlo settings
☒	☐	For hierarchical and complex designs, identification of the appropriate level for tests and full reporting of outcomes
☒	☐	Estimates of effect sizes (e.g. Cohen's *d*, Pearson's *r*), indicating how they were calculated

Our web collection on statistics for biologists contains articles on many of the points above.

Software and code

Policy information about availability of computer code

Data collection	3DHISTECH software for the acquisition of CD8 IHC images with the Panoramic 250 scanner<br>FACS DIVA software (BD Biosciences, v8.0.1) for flow cytometry experiments
Data analysis	Definiens Developer software v2.7.0. (this software is discontinued) was used to design an algorithm for CD8 IHC images<br>GraphPad Prism 7.0 and R statistical software v3.4.0 (2017-04-21) were used for for statistical analyses.<br>FlowJo, LLC. v10 for flow cytometry analysis<br>The multiGSEA function with the Camera enrichment method in the multiGSEA R package (v0.13.15) was used for gene set enrichment analysis comparing different immune phenotypes in the full ICON7 collection (n=351), with use of the Hallmark and KEGG gene set collections from the Molecular Signature Database.<br>GenomicAlignments using the R/Bioconductor package (v1.24.0) was used to quantify gene expression levels, the number of reads mapped to the exons of each RefSeq gene.<br>edgeR package (v3.30.3) was used for normalization of raw counts.<br>limma v3.32.6 (2017-09-12) Bioconductor was used to voom transformed TMN counts.<br>Random Forest package (v4.6-12).<br>ConsensusClusterPlus R package (v1.42.0) for consensus clustering<br>PAMR package (v1.55, 2014-08-27) in R to derive a classifier for the prediction of the three immune phenotypes.<br>Minfi (v1.19.0) and Illuminaio (v0.23.2) packages in R for methylation analysis<br><br>Immune subset and stromal fraction enrichment analysis for ICON7 samples were done using the online xCell cell types enrichment score tool (http://xcell.ucsf.edu/).

For manuscripts utilizing custom algorithms or software that are central to the research but not yet described in published literature, software must be made available to editors/reviewers. We strongly encourage code deposition in a community repository (e.g. GitHub). See the Nature Research guidelines for submitting code & software for further information.

Data

Policy information about availability of data

All manuscripts must include a data availability statement. This statement should provide the following information, where applicable:

- Accession codes, unique identifiers, or web links for publicly available datasets
- A list of figures that have associated raw data
- A description of any restrictions on data availability

The following datasets generated during and/or analysed during the current study from ICON7 Phase 3 trial are publically available: Images of CD8 IHC and associated digital pathology outputs (EMPIAR: https://www.ebi.ac.uk/pdbe/emdb/empiar/, accession #EMPIAR-10512); raw RNA sequencing data and clinical data (PFS) (the European Genome-Phenome Archive, accession number EGAS00001003487, https://www.ebi.ac.uk/ega/studies/EGAS00001003487). All remaining relevant data are available in the article, supplementary information, or from the corresponding author upon reasonable request. The TCGA database is accessible at this address: https://gdc.cancer.gov/ and the molecular signature database here: https://www.gsea-msigdb.org/gsea/msigdb. Source data are provided with this paper.

Field-specific reporting

Please select the one below that is the best fit for your research. If you are not sure, read the appropriate sections before making your selection.

[x] Life sciences [ ] Behavioural & social sciences [ ] Ecological, evolutionary & environmental sciences

For a reference copy of the document with all sections, see nature.com/documents/nr-reporting-summary-flat.pdf

Life sciences study design

All studies must disclose on these points even when the disclosure is negative.

Sample size	No formal sample size calculation was performed. However, with more than 300 patient samples from ICON7 phase 3 trial and additional 84 samples from an independent validation set, we believe these sample sizes are sufficient to support the key findings for the current study.
Data exclusions	Among the 370 samples from the ICON7 collection, 19 were unclassified (neither infiltrated, excluded or desert) based on the following criteria: "We confidently assigned a tumour to an immune phenotype when the probability for that phenotype exceeded 0.7 and was below 0.5 for the other two immune phenotypes. A tumour was otherwise considered unclassifiable."<br>No samples were unclassified in the vendor collection.<br>In the TCGA collection, 34 samples were unclassified among 416.<br><br>Hence, we excluded these unclassified samples from further analysis.
Replication	All replicates statements are available in the figure legends.
Randomization	Randomization is not relevant to this study because we did not compare outcomes between different arms
Blinding	Blinding is not relevant to this study because we did not compare outcomes between different arms

Reporting for specific materials, systems and methods

We require information from authors about some types of materials, experimental systems and methods used in many studies. Here, indicate whether each material, system or method listed is relevant to your study. If you are not sure if a list item applies to your research, read the appropriate section before selecting a response.

Materials & experimental systems

n/a	Involved in the study	
[ ]	[x]	Antibodies
[ ]	[x]	Eukaryotic cell lines
[x]	[ ]	Palaeontology
[x]	[ ]	Animals and other organisms
[ ]	[x]	Human research participants
[ ]	[x]	Clinical data

Methods

n/a	Involved in the study	
[x]	[ ]	ChIP-seq
[ ]	[x]	Flow cytometry
[x]	[ ]	MRI-based neuroimaging

Antibodies

Antibodies used	*Antibodies used for flow cytometry:<br>- anti-human HLA-ABC-PE (Cat#560168, BD Biosciences, clone DX17, dilution 1:10)

- isotype control mouse IgG1-PE (Cat #556650, BD Biosciences, dilution 1:10)

*Antibodies used for immunohistochemistry and in situ hybridization:
- anti-human MHC-I (Abcam, #ab52922, Clone EP1395Y, dilution 1:14,000)
- anti-human CD8 (Agilent Dako, #GA623, CD8a C8/clone 144B, assay performed by Histogenex)
- Goat anti-Rabbit IgG (H+L) Secondary Antibody (ThermoFisher, #65-6120, dilution 1:10,000)

*Antibodies used for western blot:
- anti-human phospho SMAD2 (Cell Signaling, #3108, clone 138D4, Ser456/467, dilution 1/200)
- anti-human total SMAD2/3 (Cell Signaling, # 8685, clone D7G7, dilution 1:1000)
- Anti-rabbit IgG, HRP-linked antibody (Cell Signaling Technology, #7074, dilution: 1:15,000)

Validation

Validation statement for each primary antibody is provided on the manufacturer's website.
- phospho SMAD2: https://www.cellsignal.com/products/primary-antibodies/phospho-smad2-ser465-467-138d4-rabbit-mab/3108?Ntk=Products&Ntt=3108
- total SMAD2/3: https://www.cellsignal.com/products/primary-antibodies/smad2-3-d7g7-xp-rabbit-mab/8685?Ntk=Products&Ntt=8685
- anti-human HLA-ABC-PE, BD Biosciences: https://www.bdbiosciences.com/sg/applications/research/stem-cell-research/cancer-research/human/pe-mouse-anti-human-hla-abc-dx17/p/560168
- anti-human MHC-I, Abcam: https://www.abcam.com/hla-a-antibody-ep1395y-ab52922.html
- anti-human CD8, Agilent: https://www.agilent.com/en/product/immunohistochemistry/antibodies-controls/primary-antibodies/cd8-(dako-omnis)-76236

Eukaryotic cell lines

Policy information about cell lines

Cell line source(s)

All the ovarian cancer cell lines (59M, A2780, A2780ADR, Caov-3, Caov-4, COV318, COV362, COV362.4, COV413A, COV413B, COV434, COV504, COV644, DOR-13, EFO-27, ES-2, FU-OV-1, HCC630, HCC850, HEY, Hs38.T, IGROV-1, KURAMOCHI, MCAS, NIH:OVCAR-3, OAW28, OAW42, ONCO-DG-1, OV56, OV7, OVCA-420, OvCA-429, OvCA-432, OVCAR-8, OVCAR433, OVISE, OVKATE, OVSAHO, OVTOKO, PA-1, PE01, RKN, RMUG-S, SK-OV-3, TOV-112D, TOV-21G, TYK-nu, TYK-nu.CP-r) were obtained from the Genentech Cell Bank where cell lines were purchased from ATCC, DSMZ, ECACC, JCRB, UTSW, MD Anderson Cancer Center, Fox Chase Cancer Center and Dana Farber Cancer Institute.

The human primary normal fibroblasts CCD-18-Co (colon, CRL-1459™; ATCC, Manassas, VA), HOF (ovary, #7336; ScienCell Research Laboratories, Carlsbad, CA) and Primary human bladder fibroblast (PHBF) (bladder, PCS-420-013™; ATCC) was procured from ATCC .

A detailed list of cell line sources can be available upon requests.

Authentication

All ovarian cell lines were authenticated by short tandem repeat profiling prior to banking and SNP fingerprinting after expansion.

Cell line STR and SNP profiling
Short tandem repeat (STR) profiling. DNA was extracted from cells (Qiagen DNeasy Blood & Tissue (catalogue number 69506)), the concentration determined and normalized to 50 ng ml−1. An aliquot of each was retained for SNP genotyping to identify any sample handling errors. STR analysis was performed by a third party (Genetica DNA Laboratories Inc.) using the PowerPlex 16 HS (Promega Corporation) kit which analyses 16 independent genetic sites specific for human DNA that include the 13 CODIS loci, plus PENTA E, PENTA D and amelogenin. The resulting STR DNA profile report (including allele designations and the raw data of the alleles with their graphic profiles depicting allele peak heights and areas) was used to compare against a curated list of STR profiles.

STR authentication and comparison to reference STRs
For either SNP or STR data, we applied the Tanabe algorithm (or Sørensen similarity index)18 and computed an identity score for any pair of samples as follows: for each locus at which sample 1 and sample 2 both have called alleles (that is, where neither is a ‘no call’), we computed (1) the total number of distinct alleles seen in sample 1, (2) the total number of distinct alleles seen in sample 2, and (3) the number of distinct alleles shared by both samples. Each of the three counts was then summed across all loci, and the identity score was defined as 2 × shared/(total 1 + total 2). The identity score is 0 if and only if no common alleles are seen at any locus; it is 1 if and only if the exact same alleles are seen in both samples at all loci. Note that this approach does not assume diploid genomes or biallelic markers, nor does it require that the same set of markers be available for every pair of samples.

After comparing the query profile against all STR profiles, the match is used to categorize the reference profiles as close matches (>90%) and poor matches (80–90%) to the query STR profile.

Comparison of STR and SNP profiles
Pairwise alignment scores were calculated for 836 cell lines (Fig. 2a). Heat map colours show joint STR/SNP identity score distribution when computed from true replicate pairs (48 replicate pairs for the STR assay and 2,862 replicate pairs for the SNP assay). Identity scores are computed using the Tanabe algorithm for both 16-locus STR and 48-locus SNP genotype results. Total number of comparisons was 349,030 (348,953 non-synonymous and 77 synonymous pairs of cell lines).

Univariate distributions for 16-locus STR and 48-locus SNP identity scores and a comparison of 8-locus STR and 48-locus SNP genotype are shown in Extended Data Fig. 1. For plotting purposes, a random subset of 25,000 non-synonymous pairs is displayed. Synonymous cell line pairs are well separated from the large cluster of non-synonymous pairs, but only a subset of synonymous pairs achieve identity scores similar to those typically seen for true replicate pairs.

SNP fingerprinting
SNP genotypes are performed each time new stocks are expanded for cryopreservation. Cell line identity is verified by high-throughput SNP genotyping using Fluidigm multiplexed assays32. SNPs were selected based on minor allele frequency and presence on commercial genotyping platforms. SNP genotyping reactions were setup according to manufacturer's instructions using the single target amplification method. Genotyping was performed on the Fluidigm 48.48 Dynamic Arrays and fluorescence intensity was measured on the Biomark HD System. Data analysis was done with Fluidigm SNP Genotyping Analysis v4.0.1 with a confidence threshold of 95. All genotyping calls were manually checked for accuracy and ambiguous data points were scored as no calls.

SNP profiles are compared to SNP calls from available internal and external data (when available) to determine or confirm ancestry. In cases where data are unavailable or cell line ancestry is questionable, DNA or cell lines are re-purchased to perform profiling to confirm cell line ancestry. SNPs analysed: rs11746396, rs16928965, rs2172614, rs10050093, rs10828176, rs16888998, rs16999576, rs1912640, rs2355988, rs3125842, rs10018359, rs10410468, rs10834627, rs11083145, rs11100847, rs11638893, rs12537, rs1956898, rs2069492, rs10740186, rs12486048, rs13032222, rs1635191, rs17174920, rs2590442, rs2714679, rs2928432, rs2999156, rs10461909, rs11180435, rs1784232, rs3783412, rs10885378, rs1726254, rs2391691, rs3739422, rs10108245, rs1425916, rs1325922, rs1709795, rs1934395, rs2280916, rs2563263, rs10755578, rs1529192, rs2927899, rs2848745, rs10977980.

Link to the paper describing the method: https://www.nature.com/articles/nature14397#Sec2.

Mycoplasma contamination

All cell lines tested negative for mycoplasma contamination

Commonly misidentified lines
(See ICLAC register)

All cell lines in Genentech cell line bank have been authenticated.

Human research participants

Policy information about studies involving human research participants

Population characteristics

1) The ICON7 study: Three hundred seventy treatment naïve patient samples with epithelial ovarian cancer from mixed histology were collected from a legacy phase III trial, ICON7, which examined the effect of standard chemotherapy versus adding bevacizumab to standard chemotherapy in women with newly diagnosed ovarian cancer. The clinical characteristics of these patients were previously described in the original publication of ICON7 study (Perren, T. J. et al. A phase 3 trial of bevacizumab in ovarian cancer. N Engl J Med 365, 2484-2496, doi:10.1056/NEJMoa1103799 (2011)). Clinical information are displayed in the Source Data file.

2) An independent validation collection (n=84 including 55 primary tumours and 29 paired recurrent tumours) was procured from Cureline, Inc (Brisbane, CA, US). The clinical characteristics of these patients were previously described in our earlier publication (Ryner, L. et al. Upregulation of Periostin and Reactive Stroma Is Associated with Primary Chemoresistance and Predicts Clinical Outcomes in Epithelial Ovarian Cancer. Clin Cancer Res 21, 2941-2951, doi:10.1158/1078-0432.CCR-14-3111 (2015).

Recruitment

Patient recruitment for ICON7 was detailed in ICON7 study protocol (NCT00483782) and previously described in the original publication of ICON7 study (Perren, T. J. et al. A phase 3 trial of bevacizumab in ovarian cancer. N Engl J Med 365, 2484-2496, doi:10.1056/NEJMoa1103799 (2011)).

Ethics oversight

1) The ICON7 clinical trial enrolled patients from 11 countries and led by the U.K. Medical Research Council Clinical Trials Unit (MRC CTU). Depending on national requirements, approval by ethics committees was obtained at each clinical site, nationally, or both. The ICON7 protocol was compliant with good clinical practice guidelines and the Declaration of Helsinki. Ethics approval was obtained in all participating countries and where required in all participating centres. All patients provided written informed consent.

2) Tumour samples from Cureline, Inc. The ethical committee from Saint Petersburg City Clinical Oncology Hospital approved the study under the protocol CU-2010 Oncology 12152009. All patients provided written informed consent.

Note that full information on the approval of the study protocol must also be provided in the manuscript.

Clinical data

Policy information about clinical studies
All manuscripts should comply with the ICMJEguidelines for publication of clinical research and a completedCONSORT checklist must be included with all submissions.

Clinical trial registration

ICON7 (NCT00483782)

Study protocol

The ICON7 study protocol was compliant with good clinical practice guidelines and the Declaration of Helsinki. Ethics approval was obtained in all participating countries and where required in all participating centres. All patients provided written informed consent. The protocol, including the statistical analysis plan, is available at NEJM.org.

Data collection	Data collection on ICON7 trial was previously described in the original publication of ICON7 study (Perren, T. J. et al. A phase 3 trial of bevacizumab in ovarian cancer. N Engl J Med 365, 2484-2496, doi:10.1056/NEJMoa1103799 (2011))
Outcomes	Outcomes on ICON7 trial was previously described in the original publication of ICON7 study (Perren, T. J. et al. A phase 3 trial of bevacizumab in ovarian cancer. N Engl J Med 365, 2484-2496, doi:10.1056/NEJMoa1103799 (2011))

Flow Cytometry

Plots

Confirm that:

- [x] The axis labels state the marker and fluorochrome used (e.g. CD4-FITC).
- [x] The axis scales are clearly visible. Include numbers along axes only for bottom left plot of group (a 'group' is an analysis of identical markers).
- [x] All plots are contour plots with outliers or pseudocolor plots.
- [x] A numerical value for number of cells or percentage (with statistics) is provided.

Methodology

Sample preparation	Before staining, Fc receptors were blocked for 10 min at room temperature using FcR blocking reagent human (Cat # 130-059-901, Miltenyi Biotec, CA). Cells were stained during the blocking step with the LIVE/DEAD™ Fixable Near-IR Dead Cell (Cat #L10119, Invitrogen, CA). Then, cells were incubated at room temperature for 15 min with anti-human HLA-ABC-PE (Cat#560168, BD Biosciences, CA) or isotype control mouse IgG1-PE (Cat #556650, BD Biosciences) antibodies, washed and samples were acquired on BD LSRFortessaTM flow cytometer.
Instrument	BD LSRFortessa X-20
Software	DIVA (BD Biosciences, v8.0.1) for data collection, FlowJo,LLC. v10 for data analysis.
Cell population abundance	We did not sort cells
Gating strategy	First we gated based on physical parameters (SSC-A vs FSC-A), we then excluded doublets (SSC-H vs SSC-W, then FSC-H vs FSC-W).

- [x] Tick this box to confirm that a figure exemplifying the gating strategy is provided in the Supplementary Information.
